# The Differentiation of Metastatic Mediastinal Lymph Nodes From Benign Hypermetabolic Lesions

**DOI:** 10.7759/cureus.24884

**Published:** 2022-05-10

**Authors:** Sertan Bulut, Deniz Celik, Harun Karamanlı, Zafer Aktas, Özlem Özmen, Hakan Ertürk, Nesrin Gürçay, Çiğdem Biber

**Affiliations:** 1 Department of Pulmonology, University of Health Sciences Atatürk Chest Diseases and Thoracic Surgery Education and Research Hospital, Ankara, TUR; 2 Department of Pulmonology, Alanya Alaaddin Keykubat University, Faculty of Health Sciences, Antalya, TUR; 3 Department of Chest Diseases, Güven Hospital, Ankara, TUR; 4 Department of Nuclear Medicine, University of Health Sciences Atatürk Chest Diseases and Thoracic Surgery Education and Research Hospital, Ankara, TUR; 5 Department of Radiology, University of Health Sciences Atatürk Chest Diseases and Thoracic Surgery Education and Research Hospital, Ankara, TUR; 6 Department of Pathology, University of Health Sciences Atatürk Chest Diseases and Thoracic Surgery Education and Research Hospital, Ankara, TUR

**Keywords:** squamous cell lung cancer, endobronchial ultrasound-guided transbronchial needle aspiration, squamous cell lung cancer lymph metastasis, ebus-tbna, suvmax, pet/ct, squamous cell lung cancer metastasized lymph nodes, anthracosis

## Abstract

Background

Anthracosis may cause a positron emission tomography/computed tomography (PET/CT) false positivity in mediastinal and hilar lymph nodes. We aimed to evaluate the radiological features and the maximum standardized uptake values (SUVmax) of the mediastinal lymph nodes with anthracosis or squamous cell lung cancer metastasized.

Methodology

Patients diagnosed with anthracosis or squamous cell lung cancer with endobronchial ultrasound-guided transbronchial needle aspiration (EBUS-TBNA) between January 1, 2015, and November 15, 2020, in a tertiary hospital were enrolled. The squamous cell subtype of lung cancer was selected due to its association with tobacco use, biomass, and air pollution. Anthracosis may occur due to the same etiologic reasons.

Results

A total of 190 patients met the study enrollment criteria, of which 86 were diagnosed with anthracosis and 33 with squamous cell lung cancer lymph metastasis. Median values for short axis, long axis, SUVmax, shape features, and presence of calcification were found significantly different between the groups. In receiver operating characteristic (ROC) analysis, the SUVmax cut-off value was calculated as 6.61. With this cutoff value, the negative predictive value (NPV) was 92.5% and the positive predictive value (PPV) was 54% for differentiating anthracosis and malignant lymph nodes metastasis.

Conclusions

We conclude that the evaluation of the shape and metabolic activities of the anthracotic lymph nodes detected by PET/CT together with EBUS-TBNA granted a more accurate staging of the patients and more cancer patients will benefit from surgical treatment.

## Introduction

Indoor air pollution resulting from the use of solid fuels (wood, crop residue, animal dung, coal) for cooking and heating is a significant public health concern in developing countries where a substantial proportion of the population relies exclusively on such fuels for cooking and heating [1,2]. Biomass exposure is a condition that family members have been exposed to since childhood in many rural areas of Anatolia [3].

Anthracosis may cause difficulties in the diagnosis and staging of lung cancers, and even the positron emission tomography/computed tomography (PET/CT) scan may show false positivity in mediastinal and hilar anthracotic lymph nodes. Reactive hyperplasia, hyalinized granuloma, and necrotizing granulomatous inflammation can also cause false positives in PET/CT. In a study in which mediastinal lymph nodes were confirmed with endobronchial ultrasound-guided transbronchial needle aspiration (EBUS-TBNA), high rates of false positivity were detected for level I lymph nodes (N1) and level II lymph nodes (N2) [2,4].

Anthracosis can cause difficulties in patients being evaluated for malignancy. In the literature, some studies tried to determine the radiological features of the anthracosis structure in the mediastinal and hilar lymph nodes with thorax CT [5,6]. In addition, malignancy was detected in some anthracotic lymph nodes after at least one-year follow-up or surgical removal. This situation shows that the anthracosis structure is not a definite benignity criterion [7].

In this study, we include the patients with anthracosis or squamous cell lung cancer (SCLC) diagnosed with EBUS-TBNA in our study group, regardless of the size of lymph nodes. We aimed to evaluate the radiological features of the mediastinal lymph nodes and to find a possible radiological marker between the two disease groups by comparing the PET/CT and the maximum standardized uptake values (SUVmax) because all subjects had PET/CT examinations before EBUS-TBNA.

## Materials and methods

Study population

Our study group comprises cases hospitalized in a tertiary chest diseases training and research hospital, Health Sciences University Ankara Atatürk Sanatorium Education and Research Hospital, Turkey, between January 1, 2015, and November 15, 2020. These cases consisted of patients diagnosed with EBUS-TBNA with pre-diagnoses such as malignancy, interstitial lung disease (ILD), and tuberculosis (TB), and whose final pathological diagnosis was anthracosis or SCLC. The study group consisted of patients diagnosed with a squamous cell subtype of lung cancer due to its association with tobacco use, biomass, and air pollution. The patients' histopathological diagnosis with SCLC had lung lesions besides the lymph metastasis. The second study group consisted of patients diagnosed with anthracosis, which may occur due to the same etiologic reasons too [8,9].

Enrollment criteria for anthracosis group

Patients who were over 18 years old and gave their informed consent for using their anonymous medical data at admission were enrolled for clinical studies. The lymph node was sampled by EBUS-TBNA and diagnosed as anthracosis, with no accompanying malignancy, ILD or TB. In cases with known malignancy, anthracosis in the lymph node was sampled by EBUS-TBNA and after pathological examination, if there were no signs of malignancy in the lymph node, enrolled for this study. However, this had to be confirmed by mediastinoscopy or at least one-year radiological follow-up after systemic or local cancer treatments, where the stability of the relevant lymph nodes (no growth, shrinkage, and character change) was observed. In cases known to have ILD, the sampled lymph node by EBUS-TBNA was reported as anthracosis and no signs of ILD (especially granulomatous diseases). This condition was confirmed by mediastinoscopy or the stability of the relevant lymph nodes (no growth, no shrinkage, and no character change) was observed through at least one-year radiological follow-up. Patients who had a PET/CT before EBUS-TBNA were enrolled.

Enrollment criteria for SCLC

Only those patients were enrolled, who were over 18 years old, and who gave informed consent for using their anonymous medical data at admission for clinical studies. The lymph node was sampled by EBUS-TBNA and diagnosed as SCLC with no accompanying anthracosis, ILD, or TB. Patients who had a PET/CT before EBUS-TBNA were enrolled. 

Of 164 patients evaluated for this study, 126 had anthracosis and 38 were diagnosed with SCLC. In the anthracosis group, 40 patients were excluded because of inconsistency with enrollment criteria. In the SCLC group, five patients were excluded because of inconsistency with enrollment criteria. The remaining 119 patients (86 with anthracosis and 33 with SCLC) were included in the study.

EBUS-TBNA

For all patients, EBUS-TBNA was performed under conscious sedation with midazolam, ketamine, or propofol in an operating room. An EBUS (Fujifilm Corp., Minato City, Tokyo, Japan) was used to examine the lymph nodes and the ultrasound images were processed with a dedicated scanner. We used 22-gauge needles to sample the lymph nodes.

PET/CT

Whole-body fluorodeoxyglucose F 18 (F18-FDG) PET/CT was performed using dedicated PET/CT scanners, Siemens Biograph-6-HI-REZ (Siemens Healthineers AG, Erlangen, Germany). Patients were instructed for fasting at least six hours before the examination. After confirming that the blood glucose level was below 180 mg/dL, patients received an intravenous injection of 370-555 MBq (10-15 mCi) fluorodeoxyglucose (FDG) and rested for 60 minutes before the scan. Oral contrast material was used in all patients for better visualization of the intestinal lumens. PET data were acquired from the top of the skull to the upper thigh with the arms up position. The SUVmax corrected for body weight was computed by standard methods from the activity in the most intense voxel in the three-dimensional tumor region from the transaxial whole-body images on attenuation-corrected PET/CT images.

Pathology materials

The cytological material obtained from the lymph node sampled with EBUS-TBNA was spread by the sampling clinician on two slides, dried in the air, and some of them were placed in 10% buffered formaldehyde solution and sent to the pathology laboratory with suitable containers. One of the two smears was evaluated by staining with Giemsa and the other with hematoxylin and eosin (H&E). The Cytoblock was obtained by placing the tissue piece got by transbronchial needle aspiration (TBNA) in formaldehyde solution on a paper filter, leaving it for routine follow-up, and then embedded in paraffin; 4-5 μm thick sections were taken from the Cytoblock and were evaluated by H&E staining. On-site analysis was not performed. Additional smear was taken from each case by EBUS-TBNA for microbiological examination for tuberculosis.

Statistical analysis

Categorical data were analyzed using the chi-square test. Categorical data were expressed as the total number and percentage. Kolmogorov-Smirnov or Shapiro-Wilk test, coefficient of variation value, skewness-kurtosis values, histogram, and detrended plot graphics were used for the distribution of all continuous variables. If the data were distributed normally, the Student t-test was used for analysis. If the data were not distributed normally, the Mann-Whitney U test was used for analysis. Data were expressed as mean ± SD or median (min-max), respectively. The size and SUVmax values of the lymph nodes in the study group were analyzed with the Mann-Whitney U test and the calcification and shape properties with chi-square. The SUVmax values of the lymph nodes were evaluated by receiver operating characteristic (ROC) analysis and the area under the curve (AUC) value was calculated. Sensitivity and specificity were calculated for anthracosis and the cutoff value was determined. The negative predictive value (NPV) was calculated by the determined cutoff value. In addition, the NPV was calculated for calcified structures or oval shapes. IBM SPSS Statistics for Windows, Version 22.0 (Released 2013; IBM Corp., Armonk, New York, United States) was used for analysis. A p-value of ≤0.05 was considered statistically significant.

## Results

All patients hospitalized and EBUS-TBNA performed between January 1, 2015, to November 15, 2020, were included in the study population. Of a total of 119 patients meeting the study enrollment criteria, 86 had been diagnosed with anthracosis and 33 with SCLC lymph metastasis. In the anthracosis group, 50 patients had a history of exposure that could cause anthracosis, while 36 patients had no history of exposure. A total of 25 patients had never smoked. The baseline characteristics of the 119 patients are shown in Table 1.

**Table 1:** Patient baseline characteristics

		Total	Anthracosis	Squamous cell lung cancer lymph metastasis
Patients, (n)		119	86	33
Examined lymph nodes, (n)		212	164	48
Age (years), median (min-max)		64 (32-89)	65 (32-89)	59 (49-73)
Gender (male/female)		95/24	63/23	32/1
Smoking status (n, %)	Never	25 (21%)	23 (26.7%)	2 (6.1%)
	Ex-smoker	52 (43.7%)	32 (37.2%)	20 (60.5%)
	Current	42 (35.3%)	31 (36.1%)	11 (33.4%)
Smoking package/year (mean±SD)		31±21.5	28±22.1	37±18.6
Tuberculosis history (n, %)	Positive	5 (4.2%)	4 (4.6%)	1 (3.03%)
	Negative	114 (95.8%)	82 (95.4%)	32 (96.97%)
Exposure (n)	Coal miner	1	1	0
	Wood chips	7	4	3
	Farmer	21	15	6
	Metal shavings	13	11	2
	Housewife	19	19	0
	Other	58	36	22

A total of 212 lymph nodes were examined of which 164 were in the anthracosis group (n=86) and 48 were in the SCLC lymph metastasis group (n=33). The patients diagnosed with SCLC had lung lesions besides the lymph metastasis. All lymph nodes were evaluated with thorax CT and PET/CT. The distribution of the EBUS-confirmed lymph nodes according to the American Thoracic Society (ATS) regional lymph node system is shown in Table 2. The most evaluated lymph node was the subcarinal lymph node (n=79, 37%).

**Table 2:** Distribution of EBUS-TBNA-confirmed lymph nodes on the American Thoracic Society regional lymph node system SCLC: squamous cell lung cancer; EBUS-TBNA: endobronchial ultrasound-guided transbronchial needle aspiration

American Thoracic Society regional lymph node system	Anthracosis (n)	SCLC lymph metastasis (n)	Total (n, %)
2R	1	1	2 (0.9%)
2L	0	0	0 (0%)
4R	30	12	42 (19%)
4L	20	7	27 (12.7%)
7	61	18	79 (37%)
10R	6	1	7 (3.3%)
10L	0	0	0 (0%)
11R	13	7	20 (9.4%)
11L	34	2	36 (16.9%)
Total	164	48	212

**Figure 1:**
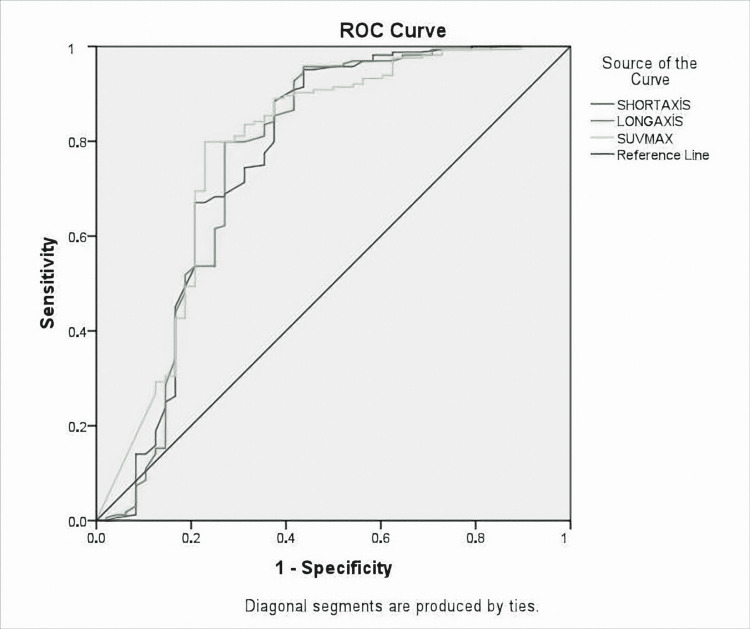
ROC analysis was performed and AUC values were compared AUC: area under the curve; ROC: receiver operating characteristic; LR: likelihood ratio

Lymph nodes were measured as the short and long axis. SUVmax values, shape features, and the presence of calcification were also noted. Radiological findings of lymph nodes are shown in Table 3. Radiological features of the anthracosis were subjected to ROC analysis and the AUC values were compared (Figure 1).

**Table 3:** Radiologic features of lymph nodes

Lymph node features		Anthracosis (n=164)	Squamous cell lung cancer lymph metastasis (n=48)	p-value
Short-axis (mm) median (min-max)		10 (4.5-21.1)	17.35 (4-33)	p<0.0001
Long-axis (mm) median (min-max)		13.25 (6-27.9 )	21.15 (5-45)	p<0.0001
SUVmax median (min-max)		3.77 (0–19.34)	9.78 (0-34.36)	p<0.0001
Calsification (n/% )	Positive	97 (59.1%)	6 (12.5%)	p<0.0001
	Negative	67 (40.9%)	42 (87.5%)	
Shape (n/% )	Oval	117 (71.3%)	25 (52.1%)	p<0.0001
	Round	47 (28.7%)	18 (37.5%)	
	Polycylic	0 (0%)	5 (10.4%)	

**Figure 2:**
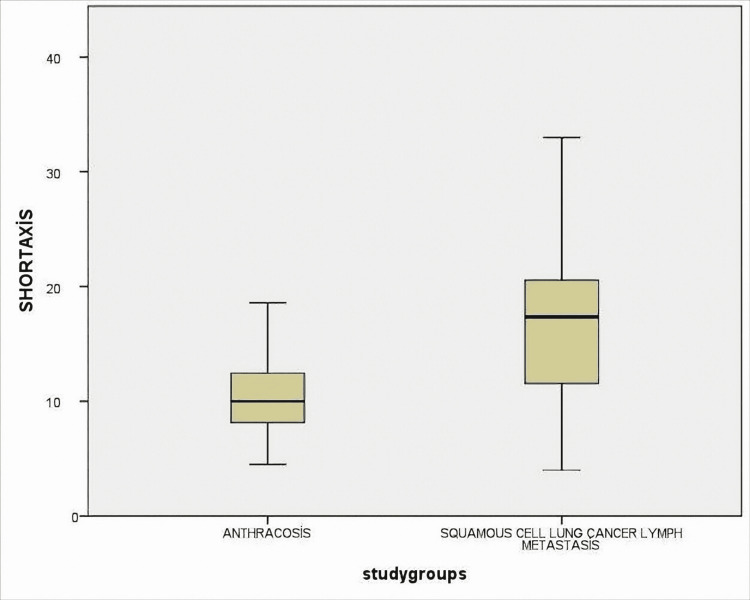
Lymph nodes were measured as short axis and compared between two groups as anthracosis and SCLC lymph metastasis The difference was statistically significant (p <0.0001) SCLC: squamous cell lung cancer

**Figure 3:**
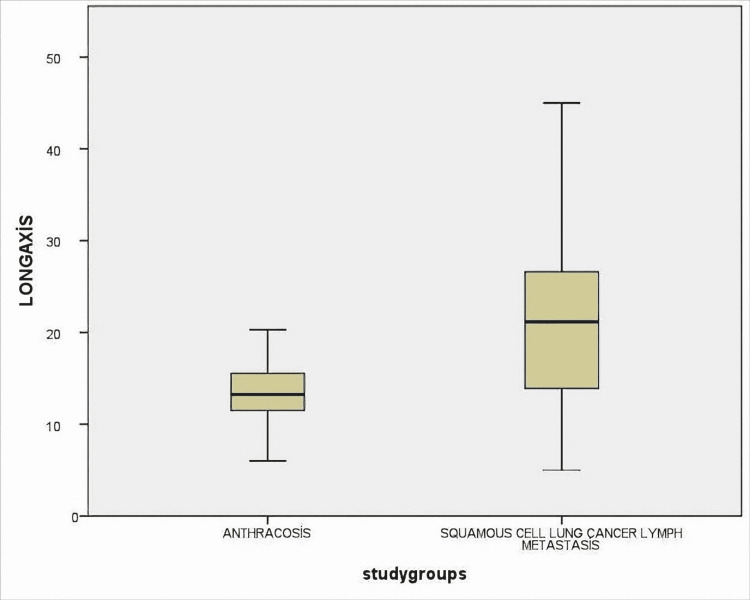
Lymph nodes were measured as long axis and compared between two groups as anthracosis and SCLC lymph metastasis The difference was statistically significant (p <0.0001) SCLC: squamous cell lung cancer

**Figure 4:**
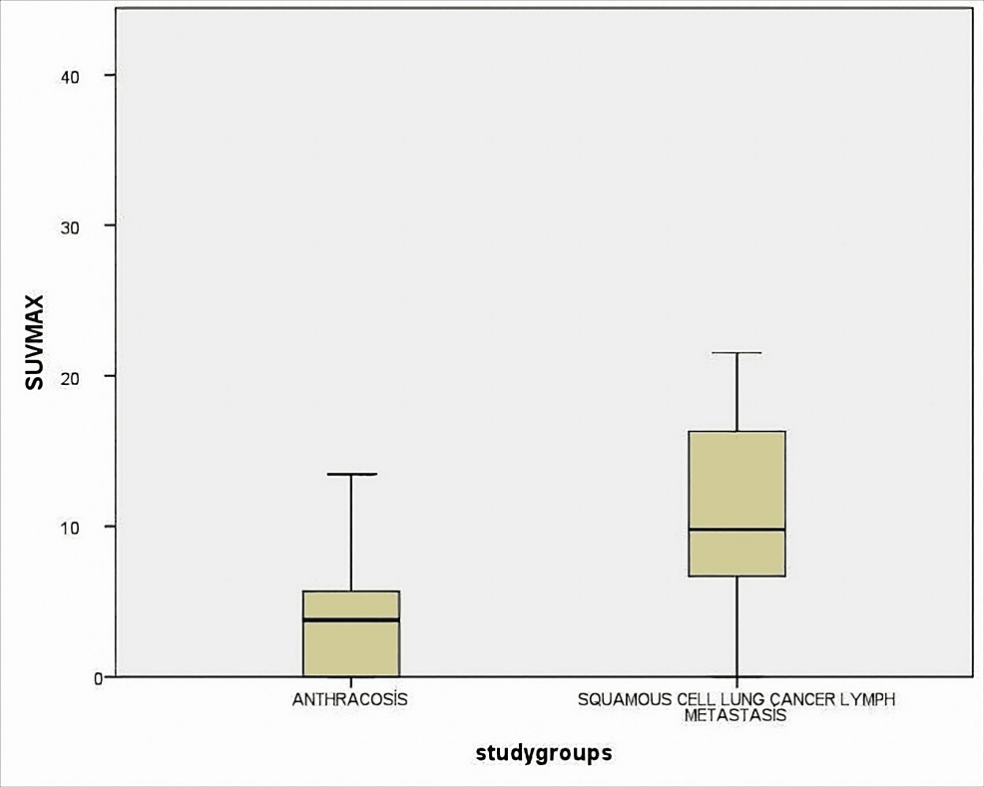
Lymph nodes were measured as SUVmax values and compared between two groups as anthracosis and SCLC lymph metastasis. The difference was statistically significant (p <0.0001) SCLC: squamous cell lung cancer

As a result of the analysis, for SUVmax, AUC=78% (0.70-0.87), for short-axis, AUC=76% (0.67-0.86), and for long axis, AUC=76%, (0.67-0.86) (Figures 2, 3, 4). All these results are statistically significant (p<0.0001). For SUVmax, while sensitivity was 80%, specificity was 57%, and likelihood ratio (LR)=3.48; the cut-off value was calculated for SUVmax and found to be 6.61.

All patients in the study were divided into two groups according to the SUVmax cutoff value of 6.61. With this cutoff value, the NPV was 92.5% and the positive predictive value (PPV) was 54% for differentiating anthracosis and SCLC lymph metastasis. The NPV was found to be 62.5% when evaluated according to the shape being oval or not. When evaluated according to whether it is calcified or not, the NPV was found to be 61.5%. Laboratory findings of the two groups were compared. C-reactive protein (CRP) and sedimentation (first hour) values were higher in the SCLC lymph metastasis group, p<0.009 and p<0.007, respectively. White blood cell, neutrophil, lymphocyte, neutrophil/lymphocyte ratio, hemoglobin, platelet, and albumin were unremarkable.

## Discussion

In our study, we evaluated patients who had a PET/CT examination and were diagnosed with EBUS-TBNA as anthracosis or metastasis of SCLC in lymph nodes, regardless of the size of the lymph node. We demonstrated that the lymph nodes in the anthracosis group were smaller in size, more oval, and more calcified and their metabolic activity was lower than in the SCLC lymph metastasis group.

Anthracosis is the black pigmentation that occurs in the bronchi of the lungs or mediastinal and hilar lymph nodes as a result of the inhalation of organic and inorganic substances due to environmental and occupational atmospheric air pollution [10,11]. The best-known cause of anthracosis is biomass exposure, and in a study conducted by Ekici et al., they found the contribution of biomass smoke to the development of chronic airway disease was 23.1% in non-smoking women [12]. Indoor coal and wood-burning may cause anthracosis and lung cancer [13]. Turkey is a country where there is exposure to biomass fuel used for various requirements as a developing country [3,12].

In the literature, according to the ATS regional lymph node system, the most studied lymph node was subcarinal, with rates of 36%, 39%, and 41.5% in some previous studies [14,5,6]. In our study, the most frequently examined lymph node with a rate of 37% was subcarinal and this was consistent with the literature data.

In the study by Kirchner et al., they compared anthracosis and other benign diseases, and they found no difference in size [6]. In another study by Kirchner et al., they took the malignant patient group and the control group as the anthracosis and found that the lymph nodes of the anthracosis group were smaller [5]. In our study, anthracosis lymph nodes were significantly smaller than the SCLC lymph metastasis (p<0.0001).

Mirsadraee et al. compared CT characteristics of patients with and without anthracosis and found that lymph nodes of the anthracosis group were calcified 70% more (p<0.05) [14]. Kirchner et al. found the rate of calcification in lymph nodes with anthracosis was 18% and the rate of being oval was 77% [5]. In our study, the rate of calcification was 59.1% and the rate of oval shape was 71.3% in the anthracosis group.

Demirci et al. compared two groups in their study. The first group was anthracosis with metabolic activity involvement in mediastinal and hilar lymph nodes. And the second group was pulmonary mediastinal lymph node metastasis in PET/CT. The mean SUVmax value of the patients with associated malignancy was 4.19 and the mean SUVmax of nonmalignant patients was 5.28. They found SUVmax values high in the group with anthracosis [15]. In their study, Köksal et al. examined SUVmax values of metastatic, anthracotic, and reactive lymph nodes, which became definite after lymph node dissection in resected non-small cell carcinoma (NSCC) cases. SUVmax values of lymph nodes in the malignant group were found significantly higher than in the other groups [16]. In our study, we found the median SUVmax values of the anthracosis group (3.77 (0-19.34)) were lower than the malignant group (9.78 (0-34.36)) (p<0.0001). In our study, 44 lymph nodes had no metabolic activity in 164 lymph nodes. When the remaining 120 patients with metabolic activity involvement were evaluated as a subgroup and analyzed, the median SUVmax value was found to be 4.44 (mean=5.74±3.32), similar to the whole anthracosis group.

Aquino et al. divided NSCC patients according to their subtypes and examined them in terms of SUVmax values. They found the mean SUVmax values for adenocarcinoma, squamous cell carcinoma, and large cell carcinoma to be 4.6, 9.2, and 7.5, respectively [17]. Because of the heterogeneous lymph node involvement among malignant groups, we included only lymph node metastasis of the squamous subtype as the control group in our study.

Bryant et al. evaluated PET/CT efficacy for pathological confirmation NSCC staging and found that if the SUVmax cutoff value is 5.3; NPV is 92% for all lymph nodes [18]. Öztürk and Güllü found the cut-off SUVmax value of 5.2 in their study [19]. With this value sensitivity was 74.8%, specificity was 84%, PPV was 82%, and NPV was 77.5%. Perigaud et al. conducted a similar study and found that the PPV and NPV were 40% and 85%, respectively [20]. Kaseda et al. found the cut-off SUVmax value of 1.7 and sensitivity was 80% and specificity was 60% [21].

Radiological features of the anthracosis group were subjected to ROC analysis and AUC values were calculated. As a result of the analysis, for SUVmax AUC=78% (0.70-0.87), for short-axis AUC=76% (0.67-0.86) and for long axis AUC=76%, (0.67-0.86). A cut-off value was calculated for SUVmax and found 6.61. All patients in the study were divided into two groups according to the SUVmax cutoff value of 6.61. With this cutoff value, the NPV was 92.5% and PPV was 54%. The NPV value is similar to the literature data. 

Our study found occupational or environmental exposure in 50 patients in the anthracosis group, but we could not find any cause in 36 patients. In addition, 23 of 86 anthracosis patients had never smoked. In our country, the migration movement from rural areas to city centers is intense and exposure to biomass fuels is evident in rural areas from birth [3]. Although we could not find a cause of anthracosis in 36 cases, we think that childhood biomass exposure may occur in these cases.

In the literature, the studies conducted with anthracosis and control groups didn’t examine the acute phase reactants and basal complete blood count. We found that CRP and sedimentation values were higher in the malignant group (p<0.009 and p<0.007). Other laboratory findings were unremarkable.

False negativity rates are reported to be quite low in the EBUS-TBNA procedure. Erol et al. conducted a study for surgical confirmation or radiological follow-up in 50 cases with anthracosis diagnosed with EBUS-TBNA. They detected malignancy in nine patients and the false-negative rate was 18% [7]. Park et al. performed mediastinoscopy in 61 anthracosis cases diagnosed with EBUS-TBNA and malignancy were detected in three patients (4.9%) [22]. In our study, we excluded the anthracosis patients with malignancy, but if we evaluate all the anthracosis cases at the beginning, we detect a definite malignancy in four of 126 cases at radiological follow-up or after mediastinoscopy (false-negative rate 3.1%).

We conclude that detecting anthracosis with EBUS for cancer staging is very likely to suggest benignity, but it is not a definite benignity criterion. The main limitations of this study are its retrospective design and the fact that it was conducted in a single center. In addition, the absence of other malignancies or benign diseases in the form of a control group is another limitation. 

## Conclusions

The shape features and metabolic activity of lymph nodes were examined with thorax CT and PET/CT guides in malignancy staging. The pathological sampling of lymph nodes with EBUS-TBNA is the gold standard in non-surgical cancer staging with low NPV. Anthracosis is common in people living in low socioeconomic conditions. We conclude that evaluation of the shape and metabolic activities of the anthracotic lymph nodes detected by PET/CT together with EBUS-TBNA should be a more accurate staging of the patients and surgical treatment will benefit more patients.
